# 316. Leveraging the Electronic Health Record to Improve OPAT Discharges at University of Colorado Hospital

**DOI:** 10.1093/ofid/ofad500.387

**Published:** 2023-11-27

**Authors:** Lorna Allen, Brian T Montague, Sandra Koehler, Heather Hallman, Nicholas L Olsen, Larissa Pisney

**Affiliations:** UCHealth University of Colorado Hospital, Aurora, Colorado; University of Colorado School of Medicine, Arvada, Colorado; University of Colorado Hospital, Aurora, Colorado; University of Colorado Hospital, Aurora, Colorado; UCHealth, Pueblo, Colorado; University of Colorado School of Medicine, Arvada, Colorado

## Abstract

**Background:**

Outpatient parenteral antimicrobial therapy (OPAT) discharges at University of Colorado Hospital (UCH) are not standardized and require a highly coordinated, multidisciplinary approach. Discrepancies and omissions on the discharge medication list have led to near miss and patient harm events during transitions of care including missed doses, wrong doses, and inappropriate cessation of IV antimicrobials. The purpose of this project was to improve patient safety and provider efficiency through streamlining and standardizing the OPAT discharge process.

**Methods:**

In September 2022 we implemented a two pronged intervention on the UCH Anschutz campus. A change in the workflow designated the Infectious Disease provider to place the antimicrobial on the discharge medication list. In addition, an OPAT navigator tool was developed in collaboration with an EPIC build team and launched in the electronic health record (EHR). The navigator utilizes discrete data entry into flow sheet rows to populate a standardized OPAT order that is automatically pulled onto the after visit summary.

**Results:**

Baseline data collected from 10/2020 - 8/2022 showed that 48% (n = 131) of OPAT patients had either incorrect or omitted orders on the discharge medication list. Post intervention data collected from 10/2022 - 3/2023 showed a mean of 98% (n= 433) of OPAT patients had the correct IV antimicrobial on the discharge medication list. Four steps were eliminated from the previous discharge workflow.

**Conclusion:**

Transitions in care are a vulnerable period for high risk OPAT patients. This project has been successful in achieving the primary aims of improved accuracy of IV antimicrobials on the discharge medication list. Continued efforts will be focused on sustainability as well as the potential for expansion to additional UCHealth sites.

**Disclosures:**

**Lorna Allen, FNP-C**, MERCK: Grant/Research Support **Brian T. Montague, DO MS MPH**, Eli Lilly: Grant/Research Support|Regeneron: Grant/Research Support

